# Eleven Novel Polymorphic Microsatellite Loci for Oval Squid *Sepioteuthis Lessoniana* (Shiro-Ika Type)

**DOI:** 10.3390/ijms141019971

**Published:** 2013-10-08

**Authors:** Satoshi Tomano, Kamarudin Ahmad-Syazni, Yukio Ueta, Kenichi Ohara, Tetsuya Umino

**Affiliations:** 1 Graduate School of Biosphere Science, Hiroshima University, Higashi-Hiroshima, Hiroshima 739-8528, Japan; E-Mails: m121065@hiroshima-u.ac.jp (S.T.); d113199@hiroshima-u.ac.jp (K.A.-S.); 2 Tokushima Agriculture, Forestry and Fisheries Technology Support Center, Naruto, Tokushima 771-0361, Japan; E-Mail: ueta_yukio_2@pref.tokushima.lg.jp; 3 Gifu Prefectural Research Institute for Freshwater Fish and Aquatic Environments, Gero, Gifu 509-2592, Japan; E-Mail: ohara-kenichi@pref.gifu.lg.jp

**Keywords:** microsatellite loci, oval squid, genetic structure, *Sepioteuthis lessoniana*

## Abstract

The oval squid *Sepioteuthis lessoniana* is one of the most economically important squid species in Japan; however, its population structure is poorly understood due to the lack of hypervariable markers. Such information is critical for managing sustainable fisheries, as well as for ensuring the existence of wild *S. lessoniana* stocks. Eleven candidate microsatellite loci were isolated from a small insert genomic DNA library. Polymorphisms in these 11 loci were screened in 24 wild individuals. The number of alleles per locus was found to range from 5 to 19 alleles, and the observed heterozygosity ranged from 0.292 to 0.958. No evidence for linkage disequilibrium was detected among all the loci. The genotypic proportions conformed to Hardy-Weinberg equilibrium, except at one locus. In conclusion, these polymorphic microsatellite loci may be used to develop a genetic framework to manage *S. lessoniana* in the future.

## 1. Introduction

The oval squid *Sepioteuthis lessoniana*, a member of the *Loliginidae* family, is distributed over a broad geographical range throughout the Indo-Pacific region, including northern Australia, central Japan, and eastward to the Hawaiian Islands [1]. In Japan, oval squids are abundant in inshore waters along the coast of western Japan and are one of the most economically important squid species in neritic fisheries.

Using allozyme markers, Izuka *et al.* [2] previously concluded that the population structure of *S. lessoniana* around mainland Japan is homogenous, in agreement with the observations of Aoki *et al.* [3] based on the sequencing analysis of the non-coding region of mitochondria. However, with the use of allozyme markers, Yokogawa and Ueta [4] also found evidence for differentiation between the Pacific Ocean and the Japan Sea populations. To date, the population structure of *S. lessoniana* over the primary biogeographic range in Japan remains unclear. A thorough understanding of the appropriate conservation units is a critical first step in addressing the fishery management of *S. lessoniana*; therefore, there is a need to identify hypervariable microsatellite loci of the species for allowing accurate evaluation of its genetic population structure.

Some researchers have proposed that *S. lessoniana* in southwest Japan is a species complex consisting of “Aka-ika”, “Shiro-ika”, and “Kuwa-ika” types that exhibit phenotypic and genotypic differences [5]. In the present study, we only evaluated the “Shiro-ika” type. We believe that this study will provide powerful genetic markers not only for studying the genetic structure of the species but also for planning the management of “Shiro-ika” *S. lessoniana* in the future.

## 2. Results and Discussion

Twenty-four unrelated individuals were collected from Mugi, Tokushima Prefecture, and the levels of polymorphism with primer sequences and repeat motifs in the 11 microsatellite loci were evaluated (Table 1). The number of alleles per locus ranged from 5 to 19 alleles. The observed heterozygosity ranged from 0.292 to 0.958, whereas the expected heterozygosity ranged from 0.368 to 0.947. Among 11 microsatellite loci, the locus *SL-SHIRO 16* deviated significantly (*p* < 0.045) from Hardy-Weinberg equilibrium after sequential Bonferroni correction, which is probably due to excess of homozygosity. The Micro-checker analysis [6] further reveals that *SL-SHIRO 16* may be affected by null alleles. However, there was no indication of allele scoring errors caused by stuttering or large allele dropout. In addition, no evidence for linkage disequilibrium was detected among possible pairwise locus comparisons.

## 3. Experimental Section

### 3.1. Development of Microsatellite Markers

To screen candidate microsatellite loci, two individuals were collected by squid jigging at Mugi, Tokushima Prefecture. A small section of the arm tissue was clipped from each specimen and stored in ethanol. DNA was subsequently extracted using the phenol-chloroform method [7], digested with *Sau3AI*, and directly ligated into the pUC19 vector. Ligated vector fragments were then transformed into competent *Escherichia coli* JM109 (Takara Bio Inc., Ohtsu, Japan). Positive colonies were detected by chemiluminescence using digoxigenin (Dig)-GT_15_ hybridization probes and the DIG Nucleic Acid Detection Kit (Boehringer, Mannheim, Germany). A total of 142 positive clones from 2352 colonies were sequenced using the ABI 3130xl Genetic Analyzer (Applied Biosystems, Foster, California, USA). Fifteen locus-specific forward and reverse primers were designed using online software Primer 3 (v. 0.4.0, Whitehead Institute for Biomedical Research, Cambridge, MA, USA) and either the forward or reverse primers were labeled at the 5′ end with FAM, HEX, or NED. Of these 15 primer pairs, only 11 were able to successfully amplify fragments within expected size ranges. Consequently, these 11 loci were characterized and deposited in GenBank (accession numbers, Table 1).

### 3.2. Primer Validation

To characterize microsatellite loci, 24 individuals were collected by a set net at Mugi, Tokushima Prefecture, Japan in 2012. Genomic DNA extracted as described above was subjected to PCR in a final reaction volume of 5 μL, consisting of 0.7 μL of ddH_2_O, 2.5 μL of KOD Buffer, 1.0 μL of dNTP, 0.1 μL of each of forward and reverse primers (10 mM), 0.1 μL of KOD Polymerase (Toyobo Co., Ltd., Osaka, Japan), and 0.5 μL of the DNA template. PCR was performed in a Mastercycler Gradient 96-Well system (Eppendorf, Hamburg, Germany) with the following amplification cycle: an initial denaturing step at 94 °C for 4 min; 30 cycles of 94 °C for 1 min, locus-specific annealing temperatures (Table 1) for 1 min, and 72 °C for 1 min; and a final extension step at 72 °C for 10 min. Next, PCR products (1 μL) were mixed with 8.8 μL of HiDi and 0.2 μL of 400HD ROX Size Standard and then sequenced using an ABI 3130xl Sequencer. Allele sizes and the number of alleles were estimated using Peak Scanner software v1.0 (Applied Biosystems) and FSTAT 2.9.3 [8], respectively. Each locus was tested for null alleles using Micro-Checker v. 2.2.3 [6]. Observed heterozygosity and expected heterozygosity were calculated using Arlequin v3.11 [9]. Other relevant parameters, including Hardy-Weinberg equilibrium and genotypic disequilibrium between pairs of loci within the population, were estimated using Genepop’007 [10].

## 4. Conclusions

The use of hypervariable microsatellite loci described in this study rendered good results when a single population was tested, suggesting that all the loci may be used for assessing the genetic diversity and the population structure of wild *S. lessoniana*, especially between the Japan Sea and Pacific Ocean. We believe that this study can not only lay a cornerstone for the investigation of accurate information regarding the stock structure and the recruitment source for managing sustainable *S. lessoniana* but also facilitate taxonomic studies of sympatric species in the future.

## Figures and Tables

**Table 1:** Characterization of 11 microsatellite loci for the oval squid *Sepioteuthis lessoniana*.

Locus	Repeat	Primer sequence (5′–3′)	T_A_ (°C)	Expected Size (bp)	N_A_	Allele Range (bp)	HO	HE	GenBank Accession No.
*SL-SHIRO 5*	(CA)4CG(CA)5(CG)3(CGCA)3	F: ACGACCTCGTCAAAAGCACT R: NED-GCTCGTCACGACTCTTGAAA	62	197	5	181–197	0.333	0.368	AB821297
*SL-SHIRO 8*	(GT)28	F: CCAACTCCGAATAAAAGCAG R: HEX-CTGTCACATCCGTGAAATGG	62	178	16	159–193	0.875	0.939	AB821298
*SL-SHIRO 10*	(CA)4TA(CA)11	F: FAM-ACAAAACGAAGGAGGGAGGT R: CATCCCACCTATTTCACAGTATCA	62	214	5	203–233	0.667	0.573	AB821299
*SL-SHIRO 15*	(GT)26AAGAAAGAGTA(GA)7	F: TTCCGCAAATTTTATGTAGCA R: HEX-TTCAGTCGAAATGAGGTAGCAG	54	215	19	189–235	0.958	0.947	AB821300
*SL-SHIRO 16*	(GTGTGA)2(GT)30	F: AAACCCGCCTGACTTCAGTT R: HEX-GGGACTCTTCCGTGACACAT	62	205	15	170–210	0.292	0.937	AB821301
*SL-SHIRO 20*	(TA)11	F: NED-CGAGTCGAGCCACACTGAAG R: TTCGGTCACCCTCGAATAAG	62	106	5	102–112	0.667	0.624	AB821302
*SL-SHIRO 22*	(TA)13	F: TCACGAGTCTTGCCTCACTG R: HEX-CCTATGTGACCGGTTTTGTTG	62	175	5	167–175	0.625	0.602	AB821303
*SL-SHIRO 23*	(CTTT)13	F: HEX-TCCTCTCTGCCACTTCCTTC R: GACAAAATGAGAGGGATACGTG	62	186	8	167–195	0.917	0.872	AB821304
*SL-SHIRO 27*	(TATG)(TATC)30	F: NED-GACAAGAGGTTTATTGGCATTGA R: ACCAACTCAAATAGTGTGCGTA	54	112	13	101–161	0.750	0.861	AB821305
*SL-SHIRO 29*	(GAAA)13	F: FAM-CTGGTGGAGGTGGGTGTTAC R: GGGTGCCTATCTGATTGCAG	54	216	10	201–233	0.875	0.863	AB821306
*SL-SHIRO 30*	(CA)12	F: TGTGTAGAAATTGTCCAAACGTC R: FAM-CAAGACACCAAGGTTTTATGGA	54	115	8	109–129	0.708	0.800	AB821307

T_A_, annealing temperature; N_A_, number of alleles; HO, observed heterozygosity; HE, Nei’s expected heterozygosity. * *p* < 0.045, significant departure from Hardy-Weinberg expectation after sequential Bonferroni correction.
